# The potential risk of *Schistosoma mansoni* transmission by the invasive freshwater snail *Biomphalaria straminea* in South China

**DOI:** 10.1371/journal.pntd.0008310

**Published:** 2020-06-08

**Authors:** DaTao Lin, Xin Zeng, Benjamin Sanogo, Ping He, Suoyu Xiang, Shuling Du, YanHua Zhang, Lifu Wang, Shuo Wan, XingDa Zeng, Ya Yang, ZhiYue Lv, YouSheng Liang, ZhuoHui Deng, Jerome Ho-Lam Hui, DongJuan Yuan, Tao Ding, ZhongDao Wu, Xi Sun

**Affiliations:** 1 Department of Parasitology, Zhongshan School of Medicine, Sun Yat-sen University, Guangzhou, Guangdong, China; 2 Provincial Engineering Technology Research Center for Diseases-vectors Control, Key Laboratory of Tropical Disease Control, Ministry of Education, Guangzhou, Guangdong, China; 3 Key Laboratory of Public Health Safety, Ministry of Education, Tropical Disease Research Center, Department of Epidemiology, School of Public Health, Fudan University, Shanghai, China; 4 Jiangsu Institute of Parasitic Diseases, Wuxi, Jiansu Province, China; 5 Guangdong Provincial Center for Disease Control and Prevention, Guangzhou, Guangdong Province, China; 6 State Key Laboratory of Agrobiotechnology, School of Life Science, The Chinese University of Hong Kong, Hong Kong Special Administrative Region, China; 7 College of Veterinary Medicine, South China Agricultural University, Guangzhou, China; University of Agricultural Sciences and Veterinary Medicine Cluj-Napoca, Life Science Institute, ROMANIA

## Abstract

Schistosomes infect more than 200 million people worldwide, and globally, over 700 million people are at risk of infection. The snail *Biomphalaria straminea*, as one of the intermediate hosts of *Schistosoma mansoni*, consecutively invaded Hong Kong in 1973, raising great concern in China. In this study, a malacological survey was conducted over a period of four years, and investigations were performed on the mechanism of susceptibility of *B*. *straminea* to *S*. *mansoni*. *B*. *straminea* was investigated in China from 2014 to 2018. Out of 185 investigated sites, 61 were positive for stages of black *B*. *straminea* (BBS), which shows pigmented spots. Twenty of the 61 sites were positive for red *B*. *straminea* (RBS), which is partially albino and red colored. Phylogenetic analyses based on *cox1* and 18S rRNA sequences demonstrated that both phenotypes were clustered with Brazilian strains. No *S*. *mansoni* infections were detected in field-collected snail. However, in laboratory experiments, 4.17% of RBS were susceptible to a Puerto Rican strain of *S*. *mansoni*, while BBS was not susceptible. The highest susceptibility rate (70.83%) was observed in the F2 generation of RBS in lab. The density of RBS has increased from south to north and from west to east in Guangdong since 2014. Five tyrosinase tyrosine metabolism genes were upregulated in BBS. Transcriptome comparisons of RBS and BBS showed that ficolin, C1q, MASP-like, and membrane attack complex (MAC)/perforin models of the complement system were significantly upregulated in BBS. Our study demonstrated that *B*. *straminea* is widely distributed in Hong Kong and Guangdong Province, which is expanding northwards very rapidly as a consequence of its adaptation to local environments. Our results suggest that *B*. *straminea* from South China is susceptible to *S*. *mansoni*, implying the high potential for *S*. *mansoni* transmission and increased *S*. *mansoni* infection risk in China.

## Author summary

*Biomphalaria straminea* is an important intermediate host for the blood fluke *Schistosoma mansoni*. *B*. *straminea* has spread in Hong Kong and in mainland China since 1973. However, whether resident snails can transmit intestinal schistosomiasis caused by *S*. *mansoni* remains unclear. Our results revealed that different types of *B*. *straminea* are widespread in cities such as Hong Kong, Shenzhen, Dongguan, Huizhou and Puning and that the distribution of the species has shifted northwards. The most important finding was that one of the phenotypes (red phenotype of *B*. *straminea*, RBS), which is highly susceptible to *S*. *mansoni*, has spread into the city of Shenzhen since 2016. The density of RBS in Guangdong Province has increased rapidly since 2014, especially since 2016. Transcriptome analysis showed that the high expression levels of ficolin, C1q, MASP-like, and membrane attack complex (MAC)/perforin models of the complement system might be associated with the mechanism of susceptibility. Our study suggested that *B*. *straminea* is susceptible to *S*. *mansoni*, implying a high potential risk of *S*. *mansoni* transmission in South China. More attention should be paid to the potential transmission of *S*. *mansoni*, and control measures should be established to prevent the spread of this snail in South China.

## Introduction

Schistosomiasis, a major neglected tropical disease, occurs as a result of an infection by parasitic trematodes of the genus *Schistosoma*. Schistosomiasis is considered one of the most important helminthic diseases of humans and thus ranks second only to malaria in causing long-term chronic human morbidity as well as high mortality rates. The World Health Organization (WHO) previously estimated that schistosomiasis caused 250 million infections and threatened nearly one-eighth of the global human population. The majority of infected persons were children[1]. This disease occurred in 78 countries of South America, Asia and Africa and was referred to as a “disease of poverty”, limiting children’s development, food production and the working ability of adults[1]. *Schistosoma mansoni* is one of the most widespread species causing human *Schistosoma* infections and is mainly distributed on the Arabian Peninsula and in Egypt, Libya, Sudan, sub-Saharan Africa, Brazil, some Caribbean islands, Suriname and Venezuela[2]. The main reason for the wide distribution of *S*. *mansoni* is its extensive adaptation to intermediate hosts in the snail genus *Biomphalaria*, while other schistosomes such as *Schistosoma guineensis*, *Schistosoma intercalatum*, *Schistosoma japonicum* and *Schistosoma mekongi* have specific adaptations to their intermediate host snails, such as *Bulinus forskalii*, *Bulinus globosus*, *Oncomelania hupensis*, and *Neotricula aperta*, respectively. Of the 35 described species of snails in the genus *Biomphalaria*, 18 can be demonstrated to become infected by *S*. *mansoni* either naturally or experimentally[2,3] and support the complete larval development of *S*. *mansoni*. Nine species were shown to be refractory to *S*. *mansoni*, and 8 species have not been tested[4]. *Biomphalaria* snails are mainly distributed in Africa, Suriname, Venezuela, the Dominican Republic, Guadeloupe, St. Lucia, and in large areas of Brazil. Therefore, the geographical locations of *S*. *mansoni* overlap with the distribution of *Biomphalaria*. Research on the biology and distributions of the *Biomphalaria* species has indicated that 4 species have expanded their native ranges widely during the last few decades[5]. Four species of neotropical origin have invaded other continents: *Biomphalaria glabrata* has invaded Egypt[3], *Biomphalaria tenagophila* has invaded the Congo (D.R.) and Europe[6,7], *Biomphalaria straminea* has invaded South China[8], and the African snail *Biomphalaria pfeifferi* has invaded Madagascar[9].

The invasion of *B*. *straminea* was first reported in Hong Kong in China in 1973[8]. In the last few decades, *B*. *straminea* has widely expanded its habitats and spread via different watercourses in southern China (nearly all of the city of Shenzhen, parts of the cities of Dongguan and Huizhou)[10,11,12,13]. However, an isolate of *B*. *straminea* (color not mentioned, collected in the city of Shenzhen) was reported to be resistant to *S*. *mansoni*[14]. The Chinese government and researchers are concerned about the rapid spread of *B*. *straminea* and the potential risk of *S*. *mansoni* transmission by *B*.*straminea* caused by the increasingly close relationship between China and South Amercia and between China and Africa. Travelers, migrants and workers of Chinese, African and South American origin coming to China might be sources of infection in China. We previously found two different phenotypes of *B*. *straminea*, red and black, in Hong Kong[15]. However, few studies have been conducted on the distribution and current infection status of *B*. *straminea* in South China. Knowledge of the biological features (i.e., isolated, morphology and susceptibility) of the different phenotypes of *B*. *straminea* is still limited. Thus, we investigated the distribution of *B*. *straminea* in South China from 2014 to 2018 and systemically evaluated the biological features of *B*. *straminea* and the potential risk of *S*. *mansoni* transmission posed by invading snails in China.

## Methods

### Ethics statement

The animal experiments were reviewed and approved by the Institutional Animal Care and Use Committee of Sun Yat-sen University (Permit No: 2016–122).

### Study sites and field investigation of *Biomphalaria* snails

To investigate the distribution and spread of *Biomphalaria* snails in Guangdong cities, a systematic field survey was conducted by Sun Yat-sen University and the Guangdong Province Centers for Disease Control and Prevention (CDC) from 2014 to 2018. *Biomphalaria*-like snails were collected close to riverbanks in Hong Kong, Shenzhen, Dongguan, Huizhou and Puning cities. We first selected the habitat areas where *B*. *straminea* snails have been reported and then expanded the investigation areas to include unknown sites from south to north. To investigate the current distribution of *B*. *straminea*, we investigated snails in spring, summer and autumn every year. Sampling was quantitative, and more than two researchers searched a 100–200 m length of the water body for 30–60 min and collected *Biomphalaria* snails. Snail density was calculated using 0.1 m^2^ frames of iron wire. The snail density at each site was measured three times, and the measurement locations at the same site were placed approximately 10 m from each other. We collected 100 snails from each site and captured all snails from the sites with fewer than 100 snails. Then, live snails were transported to the Department of Parasitology at Sun Yat-sen University for further laboratory studies. The number of *Biomphalaria* snails collected and the GPS coordinates of all surveyed sites were recorded. The diameter of the shell, diameter of the umbilicus, height of the shell and height of the last whorl were measured using a Vernier caliper. We used at least 30 snails in each group.

### Laboratory investigations of captured *Biomphalaria* snails

Morphological observation, laboratory cultivation, hybridization and susceptibility tests of both phenotypes of *B*. *straminea* were performed as described in our previous study[15]. The snails were fed sterile food and maintained at 25 to 27°C with an 80% relative humidity (RH) and a 14:10 h (L:D). The shells were removed from the snails and cleaned with 0.7% sodium hypochlorite. The soft tissue was dissected to reveal the reproductive organs in order to count the number of prostate diverticula under a stereoscopic microscope. We used at least 30 snails in each group. To investigate the phenotypic relationships between red *B*. *straminea* and black *B*. *straminea*, crossing and selfing were performed, and the ratio of the two phenotypes in the resulting offspring was calculated after separate rearing. To identify the highly susceptible *B*. *stramine*a snails, a selfed snail was infected by 10 miracidia, and its infected offspring were subjected to the same experiments until a highly susceptible *Biomphalaria* species was obtained. We used 24–30 snails for susceptibility test in each group.

### Molecular identification of *B*. *straminea*

After removing the shells from the snails, genomic DNA was extracted using the Hipure DNA Mini Kit (Magen, China) as previously described[15]. Genomic DNA was extracted separately from approximately 30 mg of head-foot (for phylogenetic analysis) or total snail (for parasite detection) tissue. Each sample was individually crushed using a bead mill in an Eppendorf tube with 1-mm-diameter inox beads (Qiagen, Germany). After removing the beads, 1 ml of STE extraction buffer, 10 μl of SDS buffer and 10 μl of proteinase K were added to the tube. Samples were heated at 55°C for 1 h. Whole DNA was precipitated with 250 μl of cold absolute ethyl alcohol and centrifuged at 100 g for 15s. After these steps, the total nucleic acid was washed by GW1 and GW2 buffers step by step and resuspended in 50 μl of AE buffer heated to 65°C. The subsequent DNA quality and quantity examinations were performed on a NanoDrop instrument (Thermo Scientific, America). Total DNA was stored at 4°C for further study. The 18S rRNA primer set was used: forward 5'-CGCCTGTTTATCAAAAACAT-3' and reverse 5'-CCGGTCTGAACTCAGATCACGT-3'. The *cox1* primer set was used: forward 5'-GGTCAACAAATCATAAAGATATTGG-3' and reverse 5'-TAAACTTCAGGGTGACCAAAAAATCA-3'. These 2 markers were amplified under the same PCR cycling conditions for phylogenetic analysis: denaturation at 94°C for 5 min, 30 cycles of 94°C for 50 s, 55°C for 50 s, 72°C for 50 s, and final extension at 72°C for 10 min.

### Phylogenetic analyses of *B*. *straminea*

The *cox1* and 18S rRNA sequences obtained from sequencing and GenBank were aligned and concatenated using MEGA7[16,17]. The sequences were used for the phylogenetic analysis performed by the neighbor-joining method in MEGA7[17]. Nodal support was evaluated using 1000 nonparametric bootstrap replicates.

### Parasite detection

*B*. *straminea* snails were dissected to observe the sporocysts of *S*. *mansoni* under a stereoscopic microscope. To detect *S*. *mansoni*, 18S rRNA was used as a reliable and sensitive molecular method. DNA extraction was performed as above described. The PCR amplification conditions were as previously described[15]. The *S*. *mansoni* 18S rRNA primer set was used[18]: forward 5'-GATCTGAATCCGACCAACCG-3' and reverse 5'-ATATTAACGCCCACGCTCTC-3'. The following PCR cycling conditions were used for parasite detection: denaturation at 94°C for 5 min, 40 cycles of 94°C for 50 s, 55°C for 50 s, 72°C for 50 s, and final extension at 72°C for 10 min. Laboratory cultured *B*. *straminea* snail genes for PCR native control were used and we used adult worms of *S*. *mansoni* genes for PCR positive control. The PCR products were analyzed by 1.5% agarose gel electrophoresis. PCR products were purified based on the manufacturer’s instructions provided with the TaKaRa Agarose Gel DNA Purification Kit Ver. 2.0 (TaKaRa, Otsu, Japan). The purified PCR products were sequenced by Majorbio Company (Guangzhou, China).

### Animals, release of Cercaria and parasite infections

Six-week-old (18–20 g) male BALB/c mice (specific pathogen free, SPF) were purchased from the Experimental Animal Center of Sun Yat-sen University, Guangzhou, China. *Biomphalaria* snails were exposed to *S*. *mansoni* miracidia at ratios of 1:10. The exposed snails were checked for parasite infections every two days starting 24 days post exposure. *S*. *mansoni* cercariae were released under light. Pictures of cercariae were obtained by stereomicroscopy (Leica, Germany). Male BALB/c mice were percutaneously infected with 50 cercariae of *S*. *mansoni* obtained from *Biomphalaria* snails. Liver tissues were fixed in 4% paraformaldehyde before further study. Five-millimeter paraffin sections were stained with hematoxylin and eosin (H&E) for granuloma analysis.

### RNA extraction, reverse transcription and real-time polymerase chain reaction (PCR)

Snails were collected and stored in TRIzol reagent (Invitrogen, New York, USA) at -80°C until processing. The shells were first removed. Total RNA was extracted as previously described[19]. Briefly, RNA extraction was performed by lysing total snail tissue samples with TRIzol reagent according to the manufacturer's instructions. Total RNA was quantified using a NanoDrop 2000 spectrophotometer. A total of 1.0 μg of RNA was converted to complementary DNA (cDNA) using a Thermo Scientific Revert Aid First Strand cDNA Synthesis Kit (Thermo Scientific, USA) according to the manufacturer’s protocol. Real-time PCR (RT-PCR) was performed using SYBR Green qPCR Master Mix (TaKaRa, Japan) according to the manufacturer’s protocol with a Light Cycler 480 detection system (Roche, Switzerland). RT-PCR was then performed for 17 different genes (including tyrosinase (TYR) domains, differentially expressed genes (DEGs): C1q, FREM, Bio-1, BPI-2, Bio-2, CD63 antigen and MPEP). The primers were synthesized by Sangon Biotech (Shanghai, China). The primer sequences are shown in **S1 Table**. The following RT-PCR cycling conditions were used: predenaturation at 95°C for 10 min, denaturation at 95°C for 15 s, 40 cycles of 54°C for 50 s and 72°C for 50 s and a final extension at 72°C for 10 min. RT-PCR reaction system for *B*. *straminea* was 20 μl.

### RNA extraction, library preparation, sequencing and sequence analysis

RNA extraction was performed as described above to enable differential gene expression analysis of *de novo* assembled transcriptomes. The exact RNA concentration was measured using a Qubit RNA Assay Kit in a Qubit 3.0 Fluorometer (Life Technologies, CA, USA). Then, library preparation for RNA sequencing was performed. Sequencing libraries were generated using the NEB Ultra RNA Library Prep Kit for Illumina (NEB, USA) following the manufacturer’s instructions. Briefly, mRNA was purified from total RNA using poly-T oligo-attached magnetic beads. Fragmentation was carried out using divalent cations under elevated temperature in NEB Next First Strand Synthesis Reaction Buffer (5X). The products were purified (AMPure XP system), and library quality was assessed with the Agilent Bioanalyzer 2100 system. The clustering of the index-coded samples was performed on a cBot Cluster Generation System using a HiSeq X Ten Cluster Kit (Illumina) according to the manufacturer’s instructions. After cluster generation, the library preparations were sequenced on an Illumina HiSeq X Ten platform by Frasergen Company (Wuhan, China) and 150 bp paired-end reads were generated. Before further analysis, quality control of the raw data was carried out with FraserQC (v1.2) software. We first filtered the low-quality tags and trimmed adaptors. Sequencing reads were aligned by using TopHat2 (v2.1.1) and Bowtie2 (v2.2.2) with the default parameters. According to the read-genome alignments, gene and isoform expression levels were quantified by the RNASeq by Expectation Maximization (RSEM, v1.3.0) program. The EdgeR (v3.6.8) package method was used to screen DEGs. Specifically, a fold change ≥2 was used to perform pathway enrichment analysis of DEGs.

### Statistical analysis

Linear correlations were calculated using GraphPad Prism version 5.0 (GraphPad Software, USA). Data are expressed as the mean ± standard error of the mean (SEM). The differences between the various groups were analyzed by one-way ANOVA using SPSS 19.0 software (SPSS Inc., USA). *P* < 0.05 was considered statistically significant.

## Results

### Distribution of *B*. *straminea* in South China

**Fig 1:**
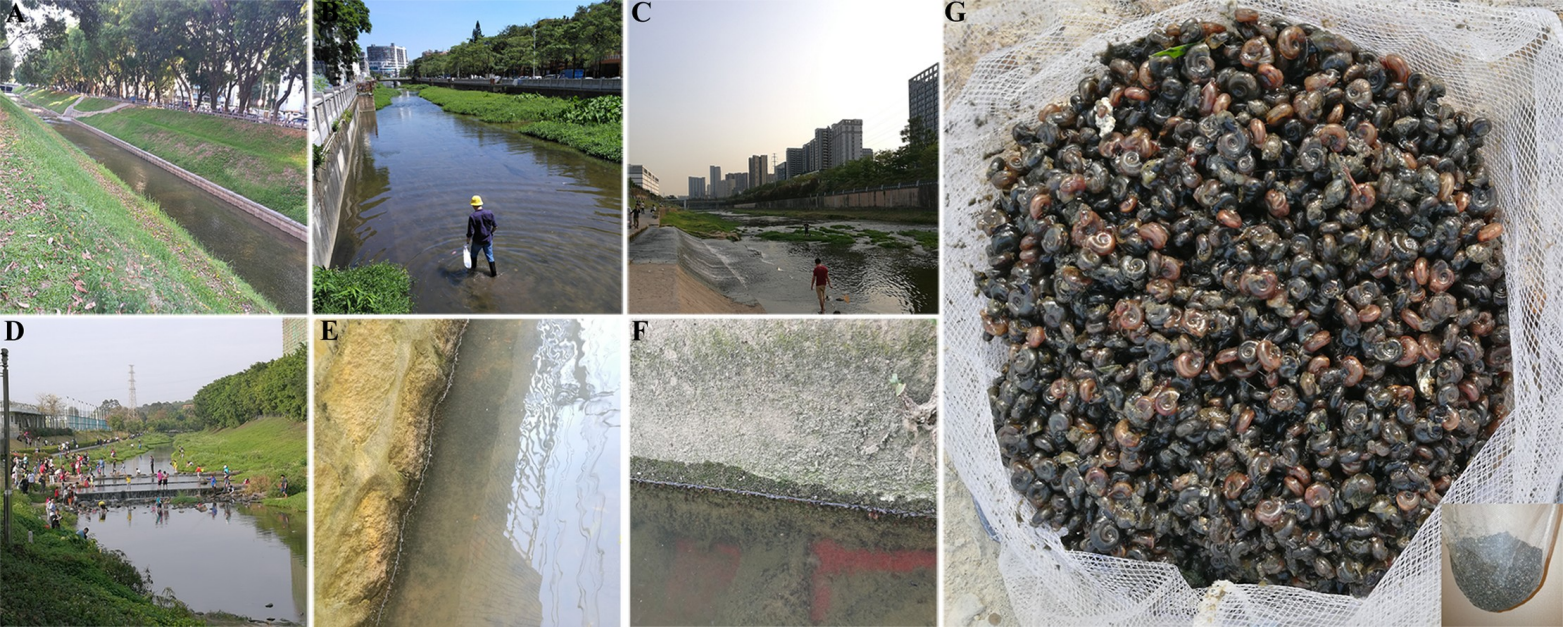
Pictures of typical *Biomphalaria* habitat in Guangdong. (**A**) River in the Xinzhou road. (**B**) Xixiang River and collecting snails. (**C**) Guanlan river and citizens playing with water. (**D**) Donghu park and citizens playing with water. (**E**) and (**F**) *Biomphalaria* snails in the field. (**G**) Captured *Biomphalaria* snails with RBS and BBS.

**Fig 2:**
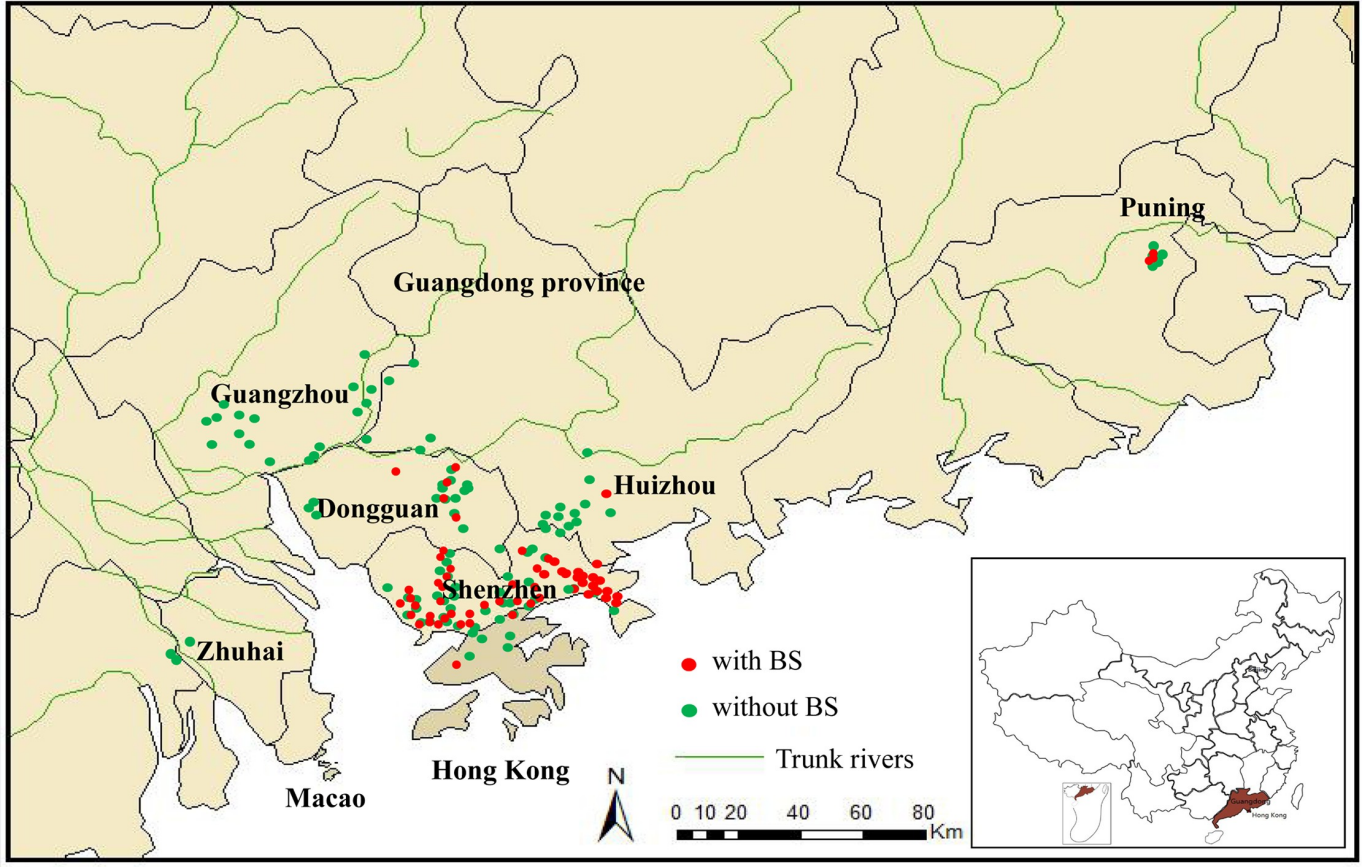
The distribution of *B*. *straminea* in Dongguan, Shenzhen, Huizhou Puning and Hong Kong Cities. Major cities and all investigated sites (positive and negative for *B*. *straminea*) were marked on the map. Green dots: negative sites; Red squares: sites found *B*. *straminea*. This map was created using ArcGIS.

Pictures representing the typical habitats of *B*. *straminea* snails in South China are shown (**Fig 1**). Among the 185 investigated sites, 163 were located in Shenzhen, Dongguan, Guangzhou, Huizhou, Puning, Hong Kong, Guangzhou and Zhuhai (**Fig 2**). Sixty-one of the sites were positive for *B*. *straminea*, including 50 sites in Shenzhen (8 in Baoan District’s rivers or reservoirs; 9 in Dapeng rivers; 4 in Futian rivers, 8 in Longgang rivers, 8 in Longhua rivers, 2 in Luohu rivers, 4 in Nanshan rivers, 5 in Pingshan rivers and 2 in Yantian rivers), 5 sites in Dongguan (4 in Guanlan/Shima rivers and 1 in an isolated site), 1 site in Huizhou/Danshui River, 3 sites in Puning and 2 sites in Hong Kong (**S1 Data**). Most of the *B*. *straminea*-positive sites were distributed within the river networks, especially the three main watercourses in Guangdong Province. *B*. *straminea* may spread from the Shenzhen River (near Hong Kong SAR) to Shenzhen, Dongguan and Huizhou in three directions from south to north and finally reach the main stream of the Dong River by the flow of the Guanlan/Shima River and Danshui River. Some sites had been reported[20,21,22,23], like Donghu Park, Tuyang, Dasha river or other sites but not mentioned in previous study, but we also found *B*. *straminea* snails at those sites or we found sites existing snails close to the reported sites. In addition, 15 isolated sites were found: 9 on Dapeng Peninsula (Shenzhen); 2 in Baoan (Shenzhen), 1 in Shilongkeng Reservoir (Dongguan) and 3 in the city of Puning.

**Fig 3:**
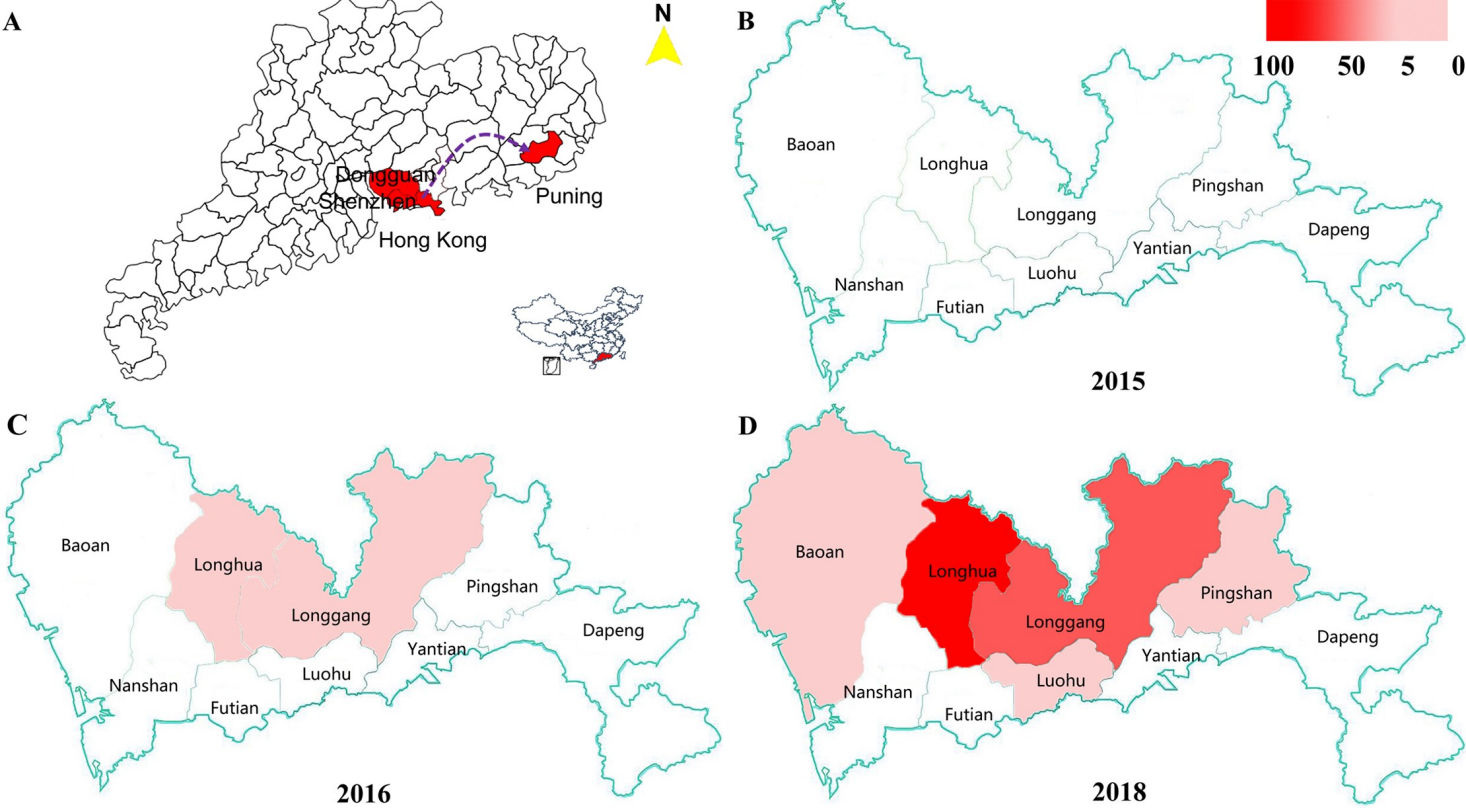
Fast expanding and distribution of red *B*. *straminea* studies in Shenzhen. (**A**) maps of RBS in Guangdong province and Hong Kong area in China. (**B**) 2015 (**C**) 2016 (**D**) 2018. Red area indicates occurrence of RBS and relates to the highest density in district. These maps were created using ArcGIS.

In the field investigation in 2014, the coexistence of two phenotypes of *B*. *straminea* (red phenotypes of *B*. *straminea*, RBS and black phenotypes of *B*. *straminea*, BBS) was detected in the town of Tangxia (city of Dongguan), one of the 60 positive sites. By 2018, RBS had distributed in Dongguan, Shenzhen, Puning and Hong Kong (**Fig 3A**). No RBS were observed in Shenzhen in 2015 (**Fig 3B**). RBS were also found in our field investigation conducted in Hong Kong during 2016. In 2016, of the 61 positive sites, the coexistence of RBS and BBS was found at 7 sites, including 5 sites in the Guanlan/Shima River and 2 sites in the Shuanglong/Danshui River (**S1 Data**). BBS was the dominant form in the investigated area, where the density of snails ranged from 15/m^2^ to more 1000/m^2^. BBS could be found in river networks and several isolated sites, and RBS were mainly found distributed in the rivers in Shenzhen (**Fig 3**) including in the Longgang, Longhua, Baoan, Luohu and Pingshan districts (**S1 Data**). In 2016, RBS was found only in the Longhua and Longgang districts in the city of Shenzhen (**Fig 3C**). However, in 2018, it spread widely to other districts in Shenzhen, including some new sites in the Baoan, Luohu and Pingshan districts, where no RBS had previously been found before (**Fig 3A and 3D**). Moreover, we found that the density of RBS had significantly increased in the Longhua and Longgang districts in 2018 (**Fig 3D**) compared with 2016 (**Fig 3C**).

### Rapid spread of the red phenotype of *B*. *straminea* in Shenzhen

We first found RBS in the town of Tangxia (Dongguan) in 2014. Then, we also found it in the towns of Qiaotou and Zhangmutou in 2016 (**S1 Data**). No RBS were encountered in Shenzhen in 2015 (**Fig 3B**). However, after the first detection of RBS in the Guanlan River (Longhua, Shenzhen), in Longyuan Park (Longgang, Shenzhen) and at Shuanglong station (Longgang, Shenzhen) in 2016 (**Fig 3C**), RBS widely spread into Luohu, Pingshan and Baoan districts in Shenzhen by 2018 (**Fig 3D**). Additionally, some new sites in the Pingshan and Baoan districts were positive for RBS, where we did not find any red-phenotype snails before 2017. Importantly, the density of RBS greatly increased in the Longhua and Longgang districts until 2018 (**Fig 3D**). In addition, we found two sites with existing RBS in the city of Puning in 2018, which were located far from the city of Shenzhen city in eastern Guangdong Province (**Fig 3A**).

### The density of red *B*. *straminea* sharply increased in Guangdong Province from 2014 to 2018

**Fig 4:**
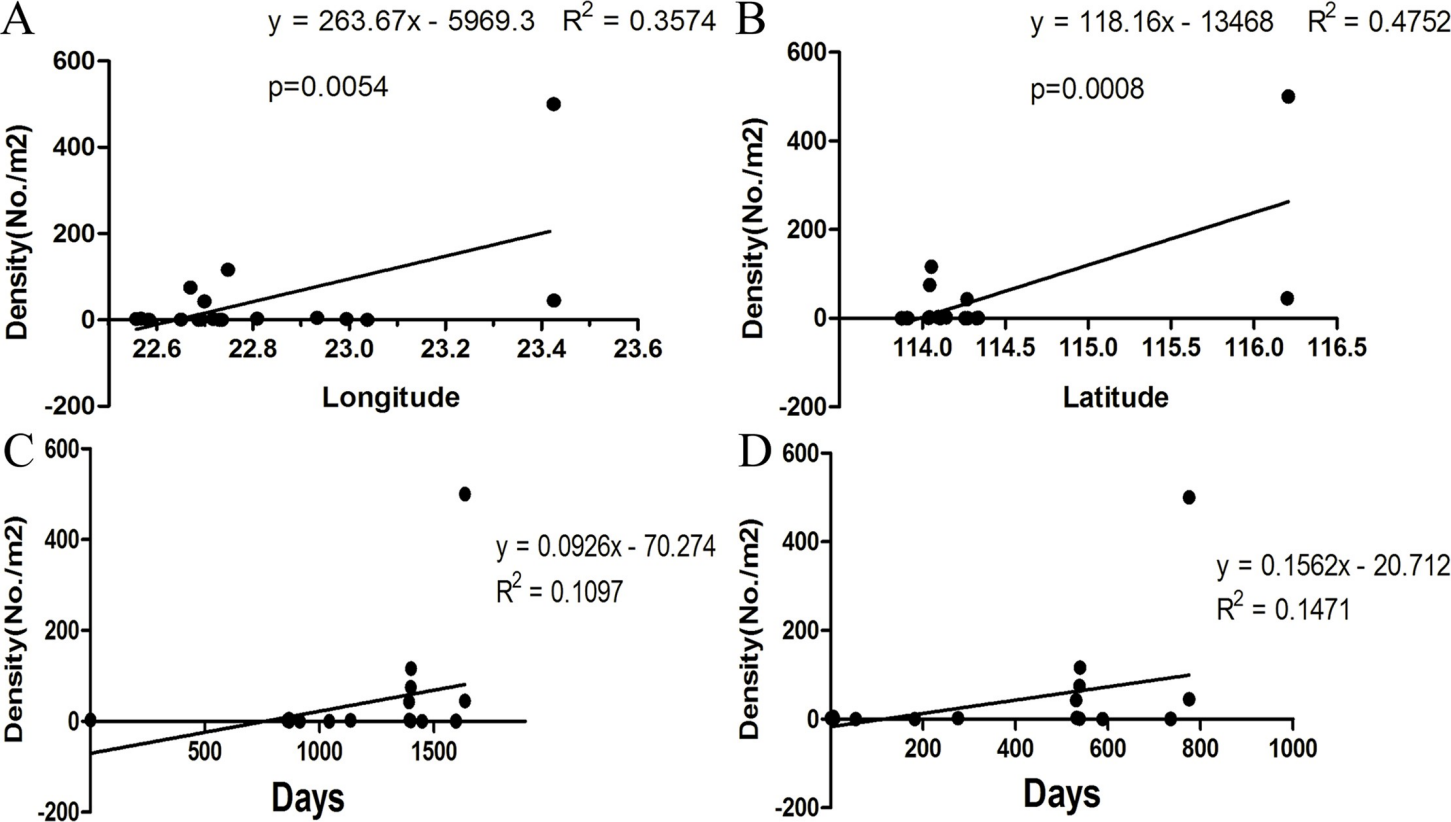
Density shifts in wild populations of red *B*. *straminea* in Guangdong province. (**A**) Relation between density and latitude. (**B**) Relation between density and longitude. (**C**) Density changes since 2014. (**D**) Density changes since 2016.

In 2014, we first found RBS in Tangxia, Dongguan, and then we analyzed its spread and distribution in South China in more detail. Correlation analysis showed that the density of RBS was significantly positively correlated with longitude (**Fig 4A**)and latitude (**Fig 4B**). The density of RBS was significantly correlated with longitude and latitude. Recently we found that RBS had already been spread to the city of Puning in the northeastern Shenzhen. This migration route showed that RBS has expanded from south to north and from west to east. In addition, the density shifts of RBS were positively correlated with time from 2014 to 2018 (**Fig 4C**). Furthermore, linear function analysis showed that the density shifts became faster from 2016 to 2018, showing a steeper slope than that from 2014 to 2016 (**Fig 4D**).

### Morphological comparison of *B*. *straminea* collected during field investigations

**Fig 5:**
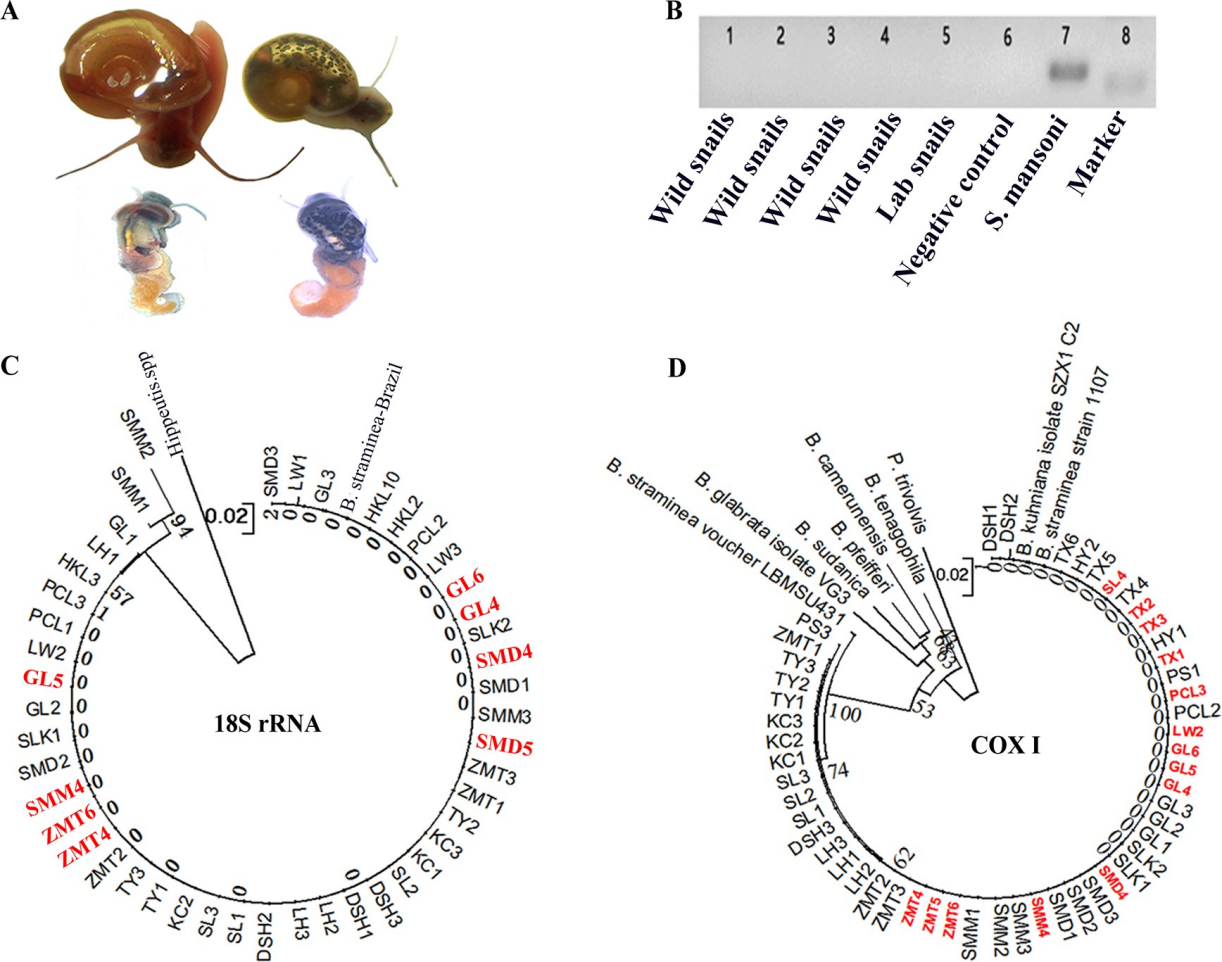
Morphological and phylogenetic features of *B*. *straminea* collected in fields and kept in laboratory. (**A**) Picture of *B*. *straminea* snails, upper: black colored snail; down: red colored snail. (**B**) Example of PCR amplifying *S*. *mansoni* specific 18S rRNA sequence extracted from field captured *B*. *straminea* (Lane 1–4), laboratorial cultured *B*. *straminea* (Lane 5), negative control (Lane 6) and adult worms of *S*. *mansoni* (Lane 7). Lane 8: 210 bp marker. (**C**) Neighbour-joining tree constructed based on K2P+G model for 18S rRNA sequences of *B*. *straminea* samples collected from different sites in south China. (**D**) Neighbor-joining tree constructed based on K2P+G model for *cox1* sequences of *B*. *straminea* samples collected from different sites in South China.

Prior to this study, the biological features of the two phenotypes of *B*. *straminea* (BBS and RBS) had not been clearly described[15]. To characterize the snails of both phenotypes, we constructed a laboratory environment to culture BBS and RBS snails. In contrast to *Biomphalaria glabrata*, snails with the black phenotype had black eyes and black spots on the mantles, while snails with the red phenotype were albino with pink eyes and the absence of melanin on the mantles (**Fig 5A**). Both phenotypes of *B*. *straminea* had black eyes (**Fig 5A**). The anatomical observations revealed dense black spots on the mantle of BBS and fewer black spots on that of RBS (**Fig 5A**). Thus, RBS was regarded as a partially albino snail because of its black eyes and few black spots on the mantle compared to those observed for BBS (**Fig 5A**).

**Fig 6:**
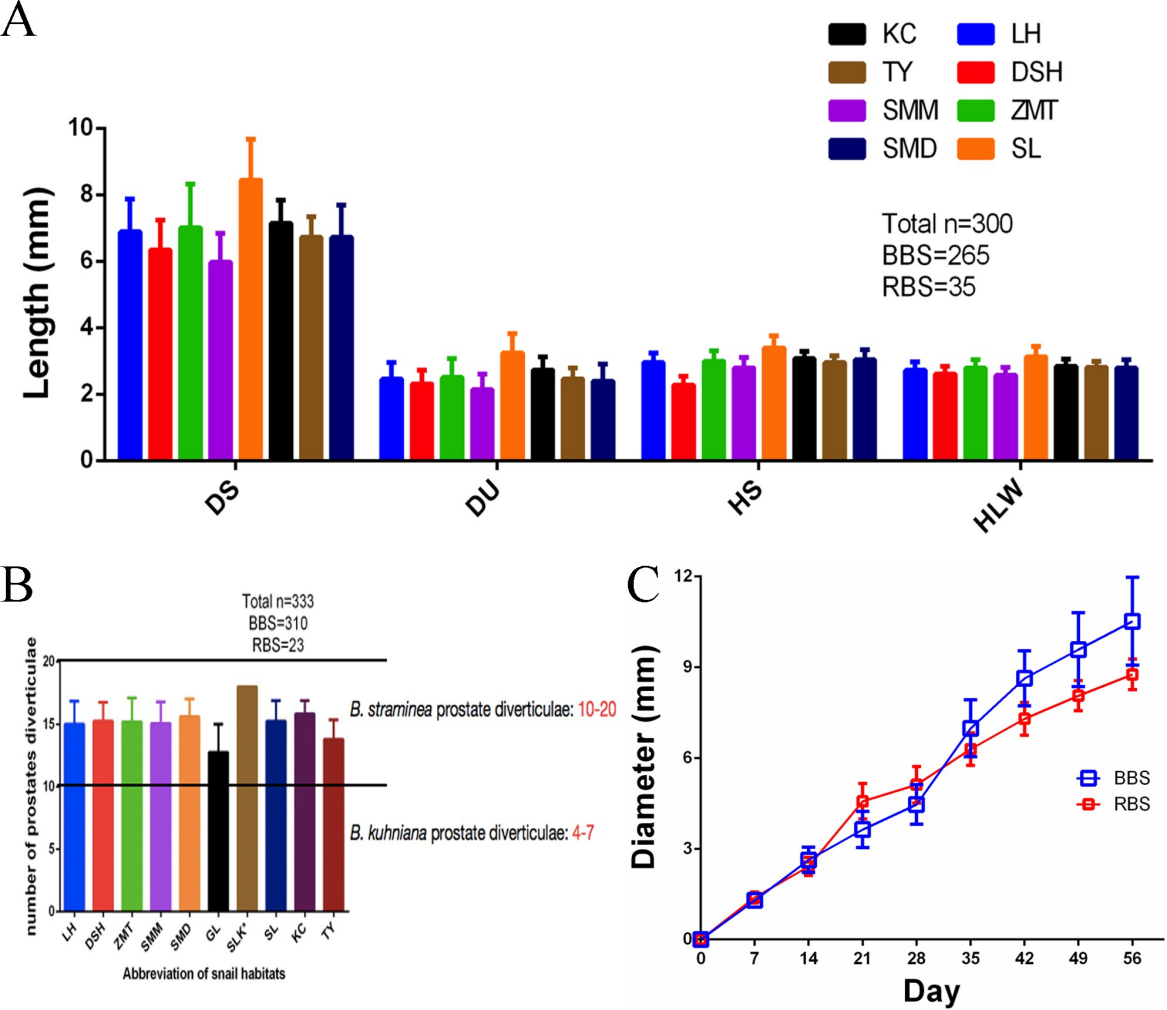
Biological features of BBS and RBS. (**A**) Configuration and size of wild *Biomphalaria* snails. (**B**) Prostate diverticula of field-captured *Biomphalaria* snails. (**C**) Growth rates between BBS and RBS reared in lab.

Three hundred snails from 8 sites were similar with respect to the size of shell, umbilicus and whorl, and the number of whorls (**Fig 6A**). The number of prostate diverticula is usually used to distinguish different species of *Biomphalaria*[24]. We found that the number of prostate diverticula of 333 *B*. *straminea* snails ranged from 12 to 17, which was consistent with the criterion for *B*. *straminea* identification (**Fig 6B**). The numbers of prostate diverticula were similar between RBS and BBS.

### Schistosome infection status of *B*. *straminea* from the field investigation

In total, 1944 *B*. *straminea* samples were dissected, and all samples (1944/1944) were negative for sporocysts of *S*. *mansoni*. A total of 90 snail DNA samples from 9 sites (10 samples in each site) were randomly selected for PCR-based detection of *S*. *mansoni*. PCR fragments of *S*. *mansoni-*specific 18S rRNA were not found in any snails from the field investigation (**Fig 5B**).

### Phylogenetic relationships of *B*. *straminea* collected from the field

To examine the phylogenetic relationships of *Biomphalaria* snails with both phenotypes (partially albino and black-spotted snails) from different sites, *cox1* and 18S rRNA were amplified and sequenced for phylogenetic reconstruction. The 18S rRNA sequences (accession: MF155555-MF155567) were highly consistent with those of Brazilian strains of *B*. *straminea* (AY030213) (**Fig 5C**). The *cox1* sequences (accession: MF179803-MF179849) of samples (including partially albino and black-spotted snails) collected in the field closely resembled (99–100%) those of Brazilian strains of *B*. *straminea* (KF926118.1) (**Fig 5D**).

### Laboratory cultivation and hybridization of the 2 phenotypes of *B*. *straminea*

**Fig 7:**
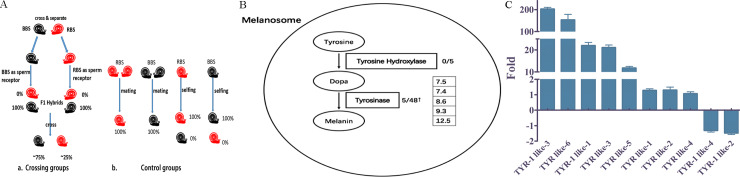
Phylogenetic relationship of *B*. *straminea* species collected from field. (**A**) Hybridization results of RBS and BBS. (**B**) Genes differentiated in melanin synthetic pathway of BBS comparing to F2RBS in transcriptomes, 0/5: 0 out of 5 tyrosine hydroxylase were up-regulated; 5/48: 5 out of 48 tyrosinase were up-regulated. Numbers in box behind represent the folds of up-regulation. (**C**) RT-PCR results of 10 different genes with tyrosinase (TYR) domain in BBS comparing to RBS.

To compare the biological features of BBS and RBS cultured in the laboratory, the growth and hybridization rates of both phenotypes were calculated. The results showed that both phenotypes had similar growth rates and grew to an approximately 10 mm diameter within 8 weeks (**Fig 6C**). The crossing results for F0 RBS and F0 BBS showed that all individuals (22/22) of the F1 generation were black spotted, while F1s crossed with each other produced approximately 25% (38/145) partially albinotic individuals (RBS) and approximately 75% (107/145) black-spotted individuals (BBS). This finding showed that the black-spotted phenotype was dominant, while the partially albinotic phenotype was recessive (**Fig 7A**). The transcriptomic results showed that tyrosine metabolism genes were defective in RBS. Five tyrosinase genes were upregulated in BBS in both transcriptomic analysis (**Fig 7B**) and RT-PCR analysis (**Fig 7C**).

### Susceptibility of both phenotypes of *B*. *straminea* snails to *S*. *mansoni*

**Fig 8:**
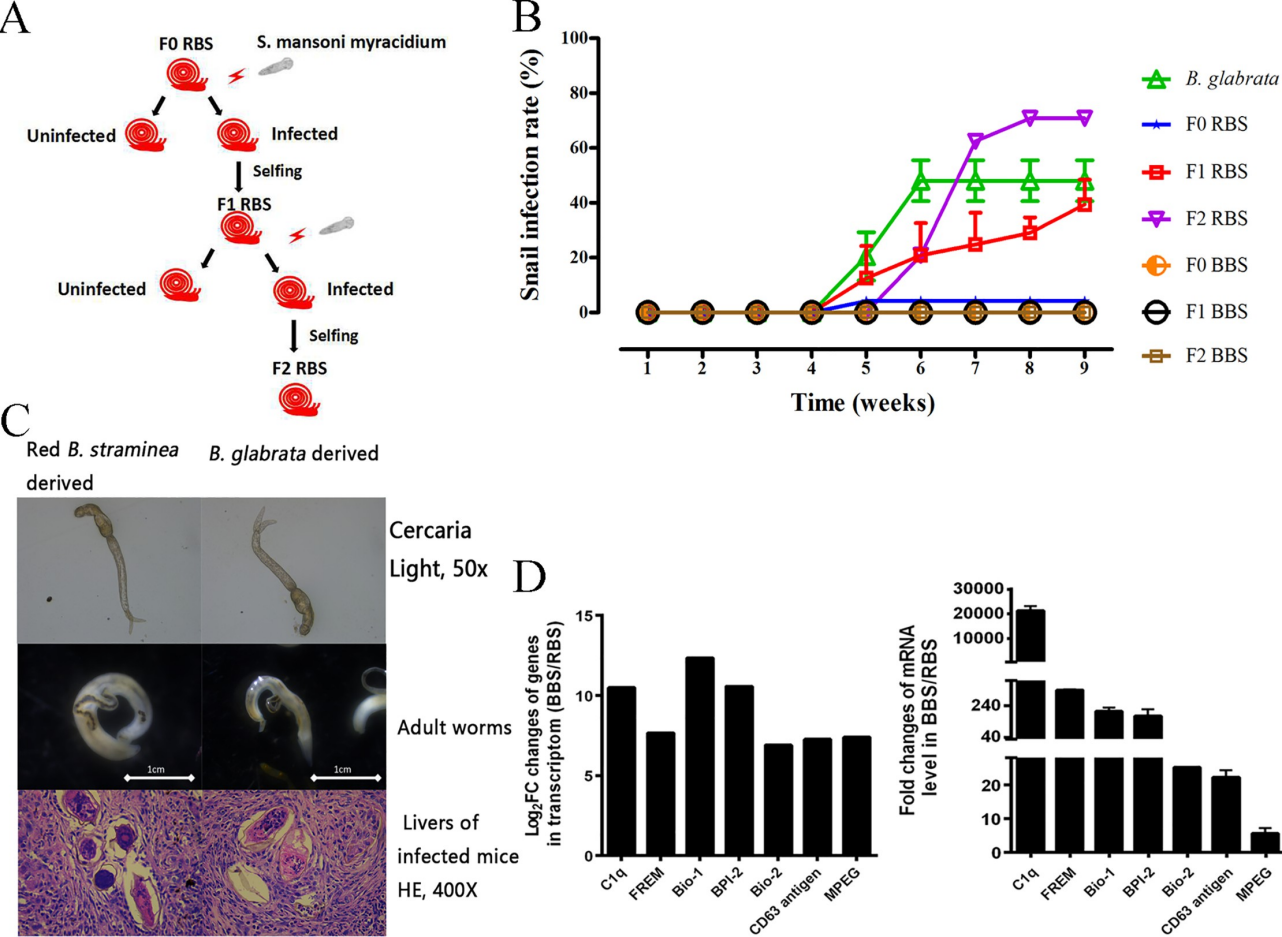
The susceptibility of *B*. *straminea* to *S*. *mansoni*. (A) Selection procedures of susceptible RBS. (B) Positive rates of *B*. *glabrata*, different generations (F0, F1, F2) of RBS and BBS infected by *S*. *mansoni*. (C) Cercariae (top), adult worms (middle) and HE stained pathological sections (mice liver) (down) of *S*. *mansoni* derived from both *B*. *straminea* and *B*. *glabrata*. (D) Analysis of differentiated expressed genes (DEGs) involved in the complementary system of BBS (black *B*. *straminea*) when comparing to F2RBS (F2 generation of selected red *B*. *straminea*). Left panel, expression levels (Log2FC) of DEGs involved in complementary system of BBS when comparing to RBS in transcriptome. Right panel, RT-PCR results of DEGs involved in complementary system of BBS when comparing to F2RBS. FREM: fibrinogen-related molecule. Bio-1: biomphalysin like protein 1, BPI-2: bactericidal permeability-increasing protein 2, Bio-2: biomphalysin like protein 2, MPEP: macrophage expressed protein.

A Puerto Rican strain of *S*. *mansoni* was used to detect susceptibility by infecting laboratory strains of partially albino (RBS) and black-spotted (BBS) *B*. *straminea* collected from Tangxia in the city of Dongguan. Highly susceptible RBS were selected by the method of artificial selection described above (**Fig 8A**). The infection rates of the F0 generations of RBS, BBS, and *B*. *glabrata* (positive control) were 4.17% (6/96), 0% (0/125) and 48.28% (56/116), respectively (**Fig 8B**). The infection rate increased to 39.58% (38/96) in the F1 generation and the highest infection rate (70.83%, 34/48) was observed in the F2 generation in laboratory tests (**Fig 8B**). There were no differences in morphological changes or the ability to infect Balb/C mice between cercariae originating from RBS and *B*. *glabrata* (**Fig 8C**). Cercariae derived from F2 RBS and *B*. *glabrata* developed into adult worms in Balb/C mice and caused similar pathological damages (as detected by HE staining), such as liver granulomatous lesions, which could be observed 8 weeks after trapping schistosome eggs within the livers of mice (**Fig 8C**). Thus, TX isolates (town of Tangxia, city of Dongguan) of RBS were susceptible to *S*. *mansoni* (Puerto Rican strain), with a positive detection rate of 4.17% (6/96), while TX isolates of BBS were resistant to *S*. *mansoni* (Puerto Rican strain). The infection rate of BBS was 0% (0/125). The increased susceptibility of F2 RBS to *S*. *mansoni* indicated that the susceptibility of *B*. *straminea* was heritable (**Fig 8B**).

### Comparison of immune genes expressed in F2 BBS and RBS transcriptomes

Among all 594 DEGs, 479 genes were upregulated and 115 were downregulated in BBS compared to RBS (**S2 Data**). The RT-PCR results for some DEGs, including C1q, fibrinogen-related molecule, biomphalysin like protein 1, bactericidal permeability-increasing protein, biomphalysin like protein 2, CD63 antigen-like and macrophage expressed protein were consistent with the transcriptomic results (**Fig 8D**). The DEGs were mostly enriched in the lysosome and phagosome pathways with enrichment factors of 14 and 12 respectively according to the KEGG pathway analysis. A detailed comparison of immune gene expression levels revealed highly expressed ficolin, C1q, MASP-like, and membrane attack complex (MAC)/perforin models in the complement system were found in BBS, while there was almost no difference in the signal transduction of pattern recognition receptors (PRRs) and their downstream targets between RBS and BBS. In addition, other immune effectors, including biomphalysin, LBP/BPI1, four FREPs, and two tetraspanins, were significantly highly expressed in BBS when compared to RBS.

## Discussion

Based on the great success of the national schistosomiasis control program "Implementation of precision control to accelerate the progress towards schistosomiasis elimination", the People's Republic of China aims to eliminate schistosomiasis by 2030. Control of the transmission of *S*. *japonicum* schistosomes in intermediate host snails remains a key strategy in the fight against schistosomiasis in the country. However, unfortunately, susceptible *B*. *straminea* (the intermediate host of *S*. *mansoni*) spread to and broke out in Guangdong Province during 2014 to 2018. Although there have been numerous investigations of the distribution of *B*. *straminea* with the black phenotype in China, whether it can transmit *S*. *mansoni* remains unclear. Globalization and China’s One Belt and One Road policy have led to major concern because susceptible snails and increasing numbers of imported patients entering the country could increase the risk of *S*. *mansoni* transmission to a critical level associated with outbreaks.

In this study, we first systematically reported two phenotypes of *B*. *straminea (*partially albinotic and black spotted) at different stages in Guangdong Province based on morphological, anatomical and molecular identification. It took only two years for *B*. *straminea* to spread from N23°02'14" to N23°25'37" and from E114°28'57" to E116°12'34". We suspect that urban construction and human activities might also be important contributors to the spread of *B*. *straminea*. Both phenotypes of snails were clustered with the Brazilian isolate of *B*. *straminea* in the phylogenetic analysis. These results were consistent with our previous findings in Hong Kong SAR in 2016[15]. However, the newly discovered partially albino strain (RBS) was neglected in previous studies[10,11,12,13]. After our first report of RBS in Tangxia, Dongguan in 2014, we explicitly delineated the current distribution of partially albino *B*. *straminea*, suggesting that it has recently spread from south to north along the Guanlan/Shima and Longgang/Danshui Rivers. RBS has already adapted to different kinds of environments and continues to spread and multiply in Shenzhen, where it may pose a great threat to public health. RBS became widespread in Shenzhen by 2018. RBS has already expanded to the city of Puning, where we found sites containing the highest density of this phenotype. Our results also showed that RBS has spread faster in the last 3 years than in previous years. RBS had already adapted, colonized and spread in Guangdong, resulting in its density sharply increasing in Guangdong Province from 2014 to 2018. All these results suggest that the red-phenotype of *B*. *straminea* may adapt to the environment and continue to multiply and spread in Guangdong, which may pose a great threat to public health in the future.

The existence of *Biomphalaria* snails in South China is debated. In 2015, Attwood reported the coexistence of *B*. *straminea* and *Biomphalaria khuniana* in South China[12]; however, other reports indicated the occurrence of only *B*. *straminea*[10,11,13]. Based on this anatomical and molecular evidence, the *Biomphalaria* snails collected in the cities of Dongguan, Huizhou, Shenzhen and Puning of Guangdong Province were demonstrated to be *B*. *straminea*. Both phenotypes of *Biomphalaria* snails were more closely related to Brazilian strains of *B*. *straminea* than to South American strains of *B*. *khuniana*. *B*. *straminea* and *B*. *khuniana* are highly similar and referred to as *B*. *straminea* complex. Furthermore, the lack of South American *B*. *khuniana* sequences available from the NCBI for assessing species differentiation has also impeded the identification of *B*. *khuniana* and B. *straminea*. However, material exports during frequent commercial contact between South China and countries in South America provide chances for neotropical *Biomphalaria* snails to invade China. Thus we cannot rule out the possibility of a biological invasion of *B*. *khuniana* in South China since *B*. *khuniana* is very similar to *B*. *straminea* in its environmental adaptation. More effective and sensitive methods are required to differentiate *B*. *khuniana* and *B*. *straminea*.

Previous studies showed that the infection rate of *B*. *straminea* with *S*. *mansoni* in Brazil was ~1.3% under laboratory conditions[25,26] and 11–24% in the field[27,28]. In our study, among the 1944 samples collected in fields in Hong Kong and in Guangdong Province, fortunately, no *S*. *mansoni* infections were detected. Regarding the susceptibility of *Biomphalaria* snails to *S*. *mansoni*, different isolates of *S*. *mansoni* and *Biomphalaria* snails showed various degrees of compatibility. In this study, we tested the susceptibility of *B*. *straminea* to only a Puerto Rican strain of *S*. *mansoni* due to limited samples available in laboratories in China. However, susceptibility tests under laboratory conditions demonstrated that RBS was susceptible to the Puerto Rican strain of *S*. *mansoni*, reaching a positive detection rate of 4.17%, while BBS was resistant. *Schistosomiasis* is an infectious disease that affects 1.8 million people in Brazil, and most federal states of Brazil are affected by *S*. *mansoni*[29,30,31]. Previous studies have demonstrated that the intermediate host *B*. *straminea* plays an important role in the transmission of *S*. *mansoni*[32,33,34]. Phylogenetic analyses clustered *B*. *straminea* from South China with Brazilian strains. According to the above results and references, we hypothesize that the *S*. *mansoni* strain from Brazil can infect the *B*. *straminea* snails from South China. Unfortunately, imported cases of *S*. *mansoni* from different countries via business persons, civil servants, worker, travelers and farmers, have been reported in recent decades[35,36,37]. Furthermore, thousands of African people with unknown *S*. *mansoni* infection status have immigrated to Guangzhou and nearby cities[36,38,39,40,41]. Therefore, more strains of *S*. *mansoni* should be investigated for their susceptibility to *B*. *straminea* in the future. Our study demonstrated that *B*. *straminea*, which has spread in Guangdong Province, may be compatible with *S*. *mansoni*, implying high risk of *S*. *mansoni* transmission and infection in China.

The highest susceptibility rate (70.83%) was observed in the F2 generation of RBS in laboratory tests. In addition, most mechanistic information previously obtained regarding intermediate host-*S*. *mansoni* interactions was based on studies of *B*. *glabrata* and *S*. *mansoni*. Our results suggested that, in addition to *B*. *glabrata*, *B*. *straminea* could also serve as a platform for studying the interactions between intermediate hosts and parasites by increasing the snail infection rate of *S*. *mansoni* via artificial selection.

The transcriptomic results indicated that defects in tyrosine metabolism might result in the partial albinism of *B*. *straminea* (RBS). This finding was consistent with the phenomenon of albinism in other invertebrates. For example, mutation of tyrosine hydroxylase in the silkworm *Bombyx mori* causes albinism in the eyes, eggs, and wings[42,43]. Thus, a difference in tyrosine metabolism might be the main reason for the appearance of partial albinism in *B*. *straminea* from South China. In addition, as an important intermediate host of *S*. *mansoni*, *B*. *glabrata* is a common model organism for researching the mechanism of susceptibility to *S*. *mansoni*. The susceptibility of *B*. *glabrata* to *S*. *mansoni* was clearly variable between the phenotypes. The well-established susceptible strain of *B*. *glabrata* serving as a host of *S*. *mansoni* exhibited albinism with a red color, while resistant strains appeared with black spots on the mantle and possessed black eyes[44,45]. Similarly, albinism in mice[46], mosquitoes[47] and *Drosophila* flies[48] leads to increased susceptibility to infections by pathogens.

Comparison of the RBS and BBS transcriptomes revealed relatively high expression of FREPs, ficolin, C1q, MASP-like, and MAC/perforin models, which are important factors in humoral responses. However, gene expression levels in TLR and TNF-TNFR signaling pathways or other effectors in the innate immune system were similar between RBS and BBS. Ficolin, C1q, MASP-like, and MAC/perforin models were found to be typical genes in the complement system of *B*. *straminea*. The complement system is the first line of defense against pathogenic intruders, comprising a group of thermolabile proteins in serum and tissue fluid. The members exhibit enzymatic activity after being activated by protease. In vertebrates, three activation pathways (the classical, lectin, and alternative pathways) converge into three broad effector pathways due to the ability of C3 convertase to exert opsonin or targeted pathogen lysis functions (by forming MACs on the pathogen surface)[49,50]. The terminal pathway of the complement produces MAC, which can mediate cell lysis to kill pathogens. Other components and split products could activate the inflammatory response and opsonization of antigen. Thus, the relatively high expression of other immune potent proinflammatory immune effectors in BBS might rapidly alert the immune system and prime subsequent immune defense against *S*. *mansoni* infection, which then contributes to the susceptibility or resistance of *B*. *straminea* to *S*. *mansoni*. The expression of the complement system and humoral immune effectors might be the main determinant of the susceptibility versus resistance of *B*. *straminea* to *S*. *mansoni*. In the future, we will explore the activity of relevant genes in determining the susceptibility of RBS to *S*. *mansoni* by detecting dynamic changes in gene expression in response to *S*. *mansoni* infections. The heritable susceptibility in different generations of RBS snails revealed the difference in how BBS and RBS defend against *S*. *mansoni*. Moreover, the results of crossing offspring of RBS and BBS suggested that restored pigmentation of *B*. *straminea* is associated with resistance to *S*. *mansoni*, indicating that the genes responsible for pigmentation might also be involved in the defense system of *B*. *straminea*.

## Conclusion

We reported on the spread and appearance of two different phenotypes of *B*. *straminea* and demonstrated that *B*. *straminea*, which is already distributed in South China, is rapidly dispersing northwards as a consequence of its adaption to the local environment. Our results suggested that *B*. *straminea* can be susceptible to *S*. *mansoni*, implying the high potential for *S*. *mansoni* transmission and increased *S*. *mansoni* infection risk in China. Thus the government and public health agencies should plan strategies to control *Biomphalaria* snails in China.

## Supporting information

S1 Table Real-time PCR primers for *B*.*straminea*. (DOCX)

S1 Data Investigated sites are shown. (XLS)

S2 Data The comparsions between BBS and RBS in transcriptomes are shown. (XLS)
