# Supplementary material for: The potential risk of Schistosoma mansoni transmission by the invasive freshwater snail Biomphalaria straminea in South China
Source: PLoS Negl Trop Dis. 2020 Jun 8;14(6):e0008310. doi: 10.1371/journal.pntd.0008310 (PMC7302743; doi:10.1371/journal.pntd.0008310)
Supplement: S1 Table — (DOCX) [file pntd.0008310.s001.docx]

**S1 Table.** Real-time PCR primers for *B.straminea*

| Gene | Forward primer  (5’→3’) | Reverse primer  (5’→3’) |
| --- | --- | --- |
| C1q | TTCCAACAACGCCATCTTGA | ATAGAGGCAATGACCCGAGA |
| Fibrinogen-related molecule | CAAGAAAGGGTCGTGGTGAA | CTCGTTGCCCAGGTAAAACT |
| Biomphalysin like protein 1 | ACTGAAGGGGAGGTAGGCTT | GTCTGCCCAGTTCACGTCAT |
| Bactericidal permeability-increasing protein | TGATCATTGGCACTGACGTT | TCCTTAGGCTGTAAGTCACCA |
| Biomphalysin like protein 2 | CTGAAGGGGAGGTAGGCTT | GCCCAGTTCACGTCATTTCT |
| CD63 antigen-like | CCGGCGCTTATGTGAAAGTG | TTAACAGTATGCCTGGGCCG |
| Macrophage expressed protein | ACTTTCAGAGGAGAGTGCTGT | ACACATTTGATGCATACTATACAGA |
| TYR-1 like-3 | TATGCGAGTTGGACTGTCGT | GGGACGTCATCATCTTGCTG |
| TYR like-6 | TGGACGATCAGTGTGGTGAA | AGGTTTTCGACGCTACTCCA |
| TYR-1 like-1 | TGTAGTAAGGCCTGTCGTGG | AGGGACGTCATCATCTTGCT |
| TYR like-3 | ACTGCATGCAAAGTACAATGAAG | AGCTAAACACAAGCTCACCG |
| TYR like-5 | ATGTATGTGTTACTGTCAACCTTG | ATGGCGCCGCTGATGTAGAC |
| TYR like-1 | ACAGGCGGTTTCAGTATCCA | ACAGGAATCATGGGCTCGAA |
| TYR like-2 | ATACACTTGACCAGCGCCAA | ACGACCAAATGCTCGCAATG |
| TYR like-4 | GTCGAGTTTGCTCTCCGTCA | TGGATTTGAGGTCTTCCGGC |
| TYR-1 like-4 | GGGATTCCTTGGCTGGCACA | CCAAAGAAGTCCGGCGTCCA |
| TYR-1 like-2 | AAGTCGTACAGCCTCAATCA | AGGGACGTCATCATCTTGCT |
| β-actin | GTCTCCCACACTGTACCTAT | CGGTCTGCATCTCGTTTTC |
